# Design, synthesis, and biological evaluation of novel quinoline-based EGFR/HER-2 dual-target inhibitors as potential anti-tumor agents†

**DOI:** 10.1039/d4ra06394e

**Published:** 2024-10-21

**Authors:** Lamya H. Al-Wahaibi, Essmat M. El-Sheref, Hendawy N. Tawfeek, Hesham A. Abou-Zied, Safwat M. Rabea, Stefan Bräse, Bahaa G. M. Youssif

**Affiliations:** a Department of Chemistry, College of Sciences, Princess Nourah Bint Abdulrahman University Riyadh 11671 Saudi Arabia; b Chemistry Department, Faculty of Science, Minia University El Minia 61519 Egypt; c Unit of Occupational of Safety and Health, Administration Office of Minia University El-Minia 61519 Egypt; d Medicinal Chemistry Department, Faculty of Pharmacy, Deraya University Minia Egypt; e Medicinal Chemistry Department, Faculty of Pharmacy, Minia University Minia 61519 Egypt; f Institute of Biological and Chemical Systems, IBCS-FMS, Karlsruhe Institute of Technology Karlsruhe 76131 Germany braese@kit.edu; g Department of Pharmaceutical Organic Chemistry, Faculty of Pharmacy, Assiut University Assiut 71526 Egypt bgyoussif2@gmail.com +20-01098294419

## Abstract

Dual targeting of EGFR and HER2 is a valid anti-cancer approach for treating solid tumors. We designed and synthesized a new series of EGFR/HER-2 dual-target inhibitors based on quinoline derivatives. The structure of the newly synthesized compounds was verified using ^1^H NMR, ^13^C NMR, and elemental analysis. The targeted compounds were tested for antiproliferative efficacy against four cancer cell lines. All the compounds had GI_50_s ranging from 25 to 82 nM, with breast (MCF-7) and lung (A-549) cancer cell lines being the most sensitive. Compound 5a demonstrated the most significant antiproliferative action. With inhibitory (IC_50_) values of 71 and 31 nM, respectively, compound 5a proved to be the most effective dual-target inhibitor of EGFR and HER-2, outperforming the reference erlotinib (IC_50_ = 80 nM) as an EGFR inhibitor but falling short of the clinically used agent lapatinib (IC_50_ = 26 nM) as a HER2 inhibitor. The apoptotic potential activity of 5a was examined, and the findings demonstrated that 5a promotes apoptosis by activating caspase-3, 8, and Bax while simultaneously reducing the expression of the anti-apoptotic protein Bcl-2. The docking studies provided valuable insights into the binding interactions of compounds 3e and 5a with EGFR, effectively rationalizing the observed SAR trends.

## Abstract 2

A series of new quinoline-based derivatives was designed and synthesised. The structures of the new compounds were validated by IR, NMR, and elemental analysis. The new compounds were evaluated as antiproliferative agents targeting EGFR, and HER2.

## Graphical abstract

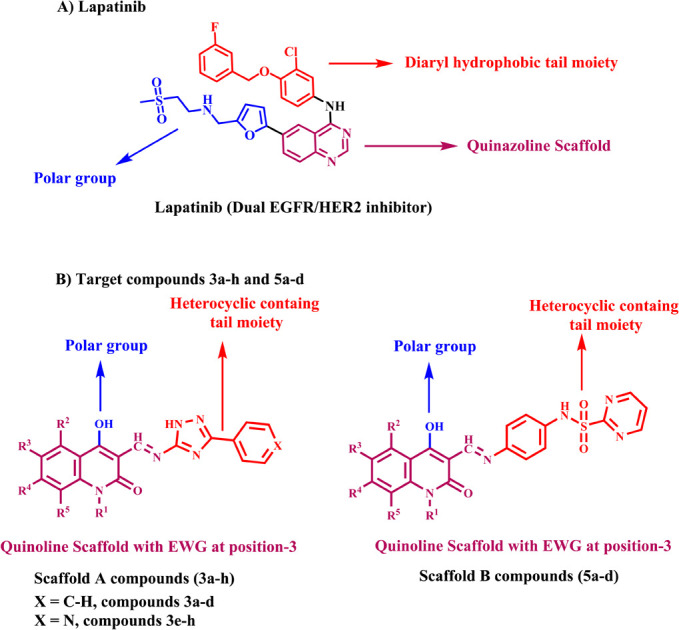

## 1. Introduction

Cancer is one of the most lethal diseases, impacting around 7 million individuals annually worldwide. Cancer is defined by the loss of control over cell proliferation, resulting in the formation of a mass of cells.^1–4^ However, cancer is frequently associated with death due to metastasis, which is the process of spreading cancer to other parts of the body and establishing additional cancerous growths.^5–7^ Research has revealed that cancer treatment methods like surgery and radiation are ineffective in the cases of spreading of tumors. As a result, numerous scientific experiments aimed at treating cancer have relied on traditional chemotherapy.^8,9^ Unfortunately, conventional chemotherapy does not distinguish between normal and damaged human cells, resulting in various side effects.^10,11^ Therefore, researchers have devised a novel approach to cancer treatment to overcome these limitations. This approach entails using specific tumor medications known as molecular targeted therapies, blocking key receptors and signaling pathways that promote tumor cell growth.^12,13^

**Fig. 1:**
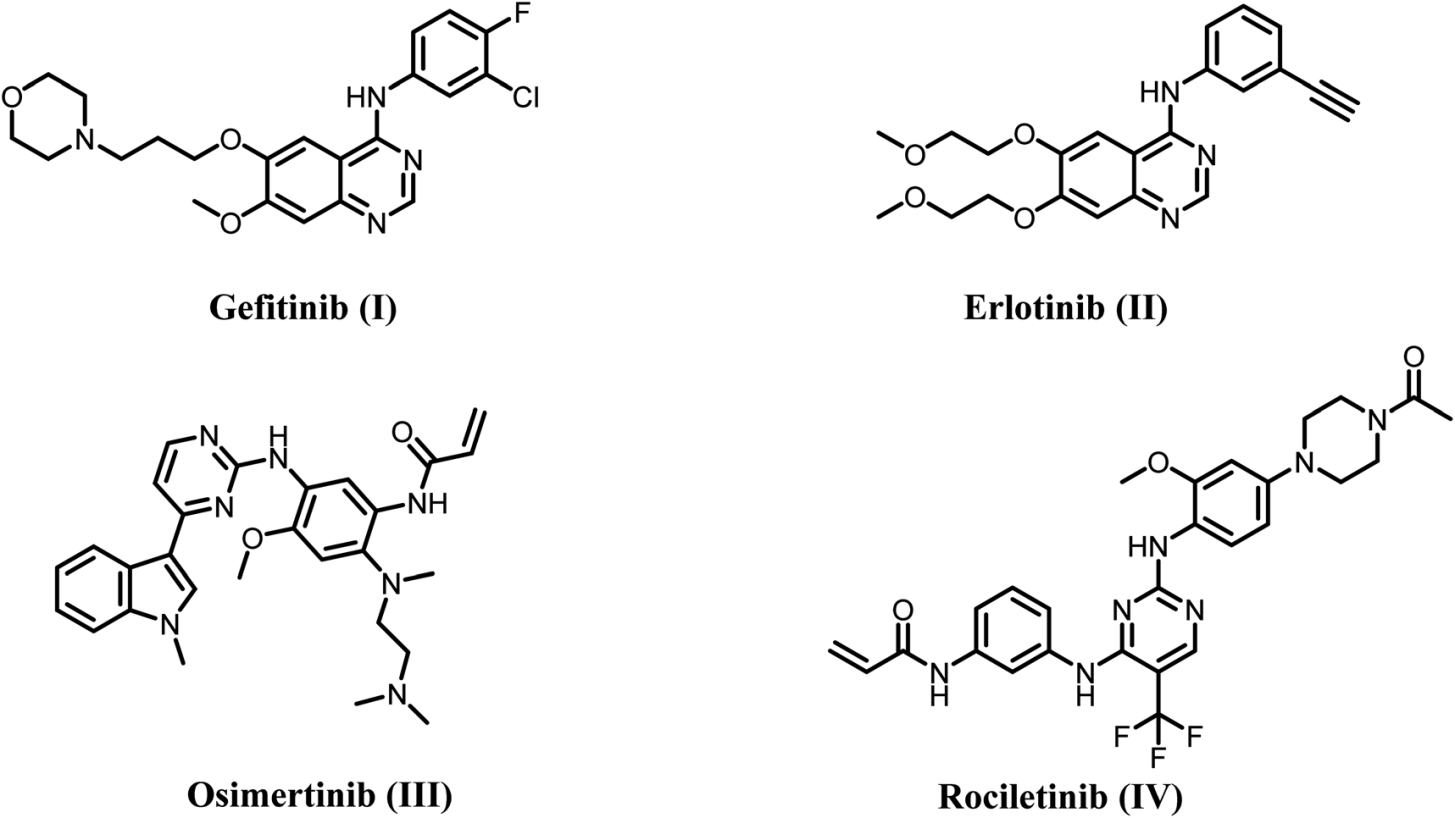
Structures of single target EGFR inhibitors I–IV.

In the body, human epidermal growth factor receptors (HERs) are a group of receptor protein tyrosine kinases (RPTKs) that help control many functions, such as cell growth, proliferation, and differentiation. Many investigations have shown that the HER protein kinase family is vital in promoting cancer advancement by affecting the release of pro-angiogenesis factors from cancer cells.^14,15^ Four closely similar isoforms of HERs have been identified, all possessing tyrosine kinase activity. These isoforms are EGFR (sometimes referred to as HER-1), HER-2, HER-3, and HER-4. Different cancer cells significantly upregulate epidermal growth factor receptor (EGFR), which plays a crucial role in cell signaling transmission and tumor behaviors.^16,17^ The use of four generations of EGFR single target inhibitors (Fig. 1), namely gefitinib, erlotinib, osimertinib, rociletinib, cetuximab, and necitumumab, has significantly advanced in both clinical and pre-clinical studies for the treatment of various cancer types, including breast cancer, bowel cancer, and non-small cell lung cancer (NSCLC).^18–21^ Unfortunately, EGFR mutations and compensatory mechanisms have significantly restricted the therapeutic effectiveness of EGFR single-target medicines.

**Fig. 2:**
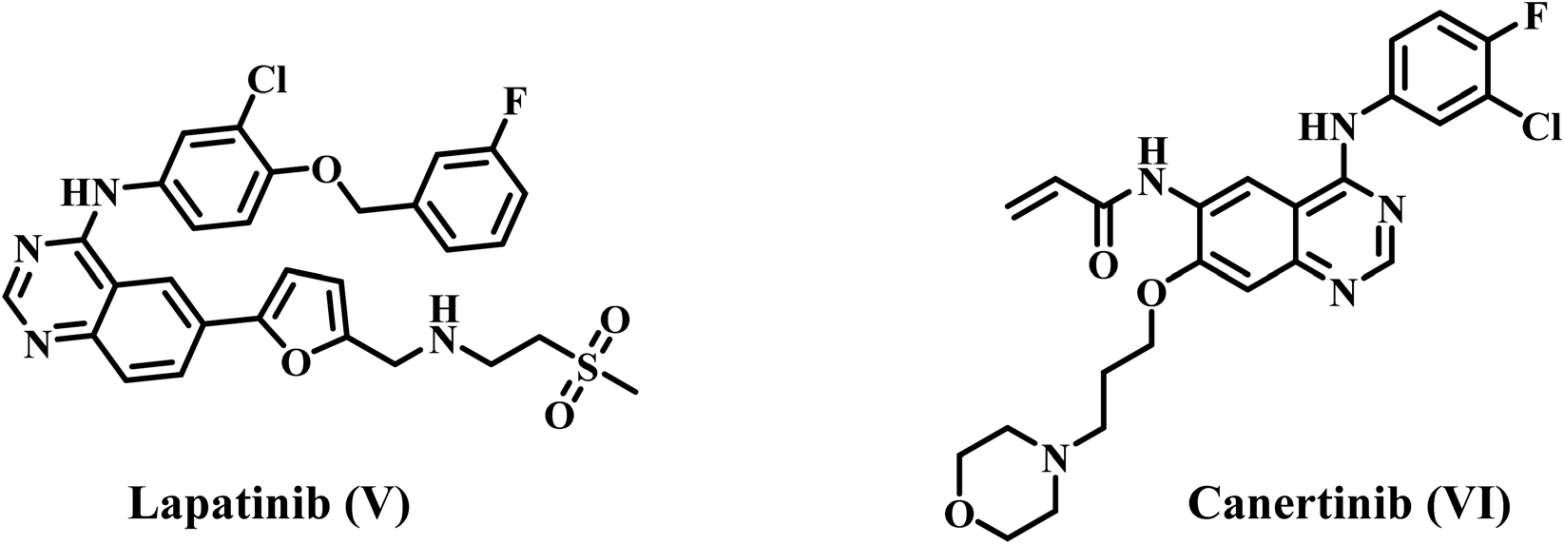
Structures of dual EGFR/HER2 inhibitors V and VI.

As a result, the development of dual inhibitors that target both EGFR and other compensatory targets has the potential to be a new therapeutic strategy to counteract drug resistance in clinical settings and merits further investigation. Studies have shown that long-term use of the EGFR inhibitor gefitinib can downregulate EGFR expression but upregulate HER2. However, anti-EGFR therapy alone can only suppress EGFR-mediated downstream signals, with minimal effect on HER-2-caused ones.^22,23^ Because of this, targeting both EGFR and HER-2 simultaneously might be an effective way to get around the resistance seen with single-agent therapy (Fig. 2).

Quinoline has been one of the most important scaffolds in drug discovery over the past few decades, particularly in cancer research. Quinoline, an N-based heterocyclic compound, has diverse biological actions. ^24,25^ Quinoline-containing compounds have significantly enhanced basicity due to the presence of nitrogen atoms. Clinical trials currently examine many anticancer drugs incorporating the quinoline structure.^26–28^ Quinoline derivatives are very good at fighting cancer through several pathways, such as blocking tyrosine kinase, blocking EGFR, and blocking mitogen-activated protein kinases, *etc.*^29,30^ Quinoline-derived anticancer drugs include bosutinib, lenvatinib, and cabozantinib, which are protein kinase inhibitors. Quinoline derivatives have shown promise in several cancer cell lines, such as those derived from the breast, colon, lung, colorectal, renal, and so on.^31–33^

Additionally, Schiff's bases are a significant category of therapeutic compounds with biological activity that has captured the interest of medicinal chemists because of their diverse range of pharmacological properties. Several researchers are synthesizing these molecules into pharmaceuticals to effectively treat diseases with the lowest toxicity and maximum efficacy.^34,35^ These predictions have provided a therapeutic approach for developing novel and potent biologically active Schiff's base derivatives. Documentation has shown that several derivatives of Schiff's base exhibit a wide range of biological activities, with anticancer properties being the most prominent.^36,37^

**Fig. 3:**
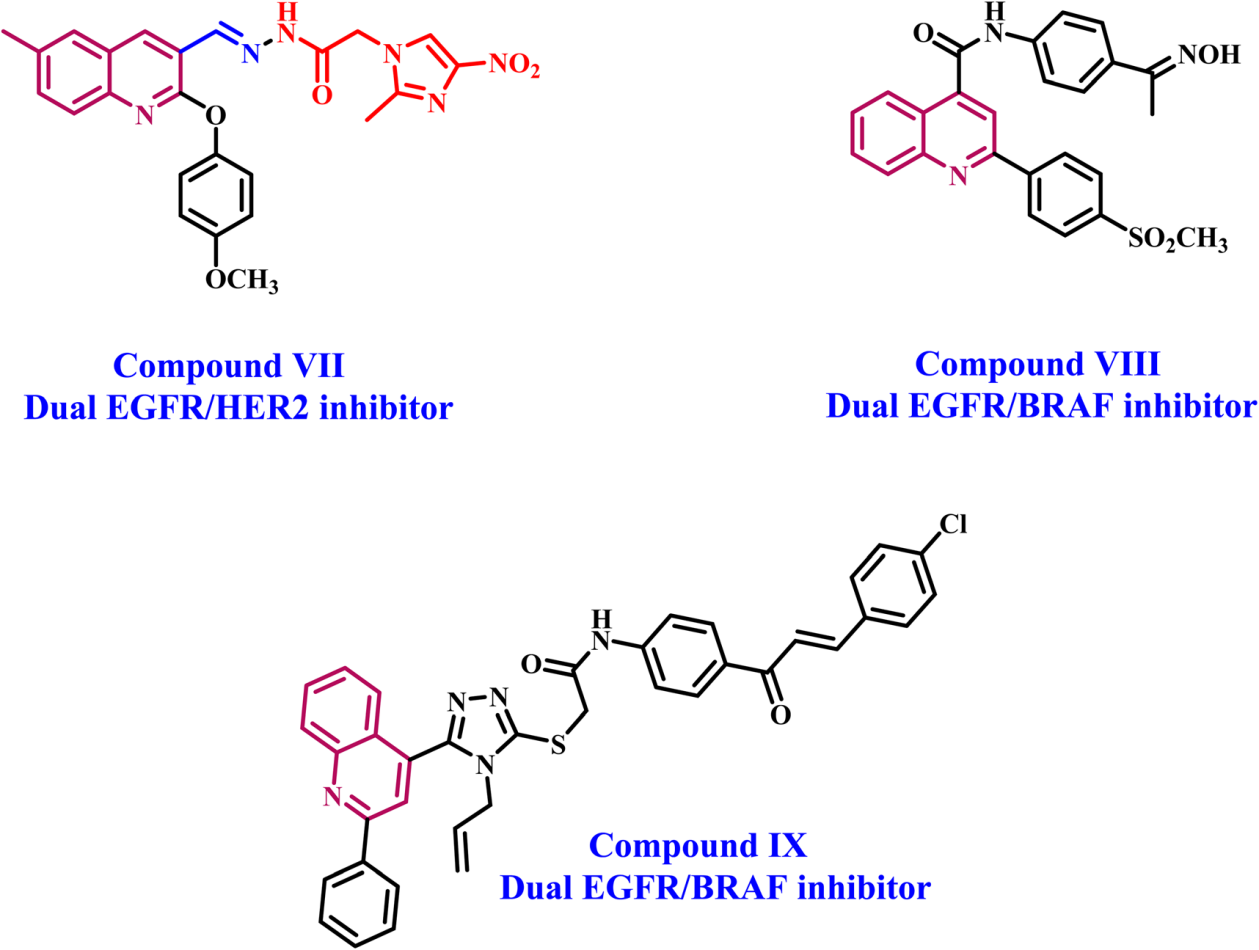
Structures of quinoline-based dual inhibitors VII–IX.

Makawana *et al.*^38^ have synthesized a series of quinoline/Schiff base-based compounds that act as anticancer agents, specifically targeting both EGFR and HER2. The results indicated that most of the compounds had potent antiproliferative effects and effectively inhibited the activities of EGFR and HER-2. Compound VII (Fig. 3) had the highest level of inhibition against EGFR (IC_50_ = 0.12 ± 0.05 μM) compared to erlotinib (IC_50_ = 0.032 ± 0.002 μM). In addition, compound 5h showed significant inhibition of HER2 with an IC_50_ value of 2.18 ± 0.08 μM, whereas erlotinib had an IC_50_ value of 0.16 ± 0.02 μM.

Our recent studies^39^ focused on developing and synthesizing novel quinoline-based compounds as potential antiproliferative agents. We evaluated the newly synthesized compounds' antiproliferative activity against a panel of four human cancer cell lines. Compound VIII (Fig. 3) was more effective than the standard drug doxorubicin against the four cancer cell lines (GI_50_ = 1.40 μM *vs.* 1.20 μM for VIII). The compound VIII was the most effective at blocking EGFR and BRAF^V600E^, with IC_50_ values of 105 ± 10 nM and 140 ± 12 nM, respectively. These values were similar to those of the standard drug erlotinib, which had IC_50_ values of 80 ± 10 nM and 60 ± 10 nM, respectively. In another publication,^40^ we describe synthesizing a novel series of quinoline-based compounds used as antiproliferative agents against EGFR and BRAF^V600E^. Compound IX (Fig. 3) had superior antiproliferative activity compared to doxorubicin (GI_50_ = 1.15 μM). It exhibited a GI_50_ value of 3.30 μM against four human cancer cell lines. The compound exhibited inhibitory efficacy against EGFR and BRAFV^600E^, with IC_50_ values of 1.30 ± 0.12 μM and 3.80 ± 0.15 μM, respectively. In comparison, the reference erlotinib had IC_50_ values of 0.08 ± 0.005 μM and 0.06 ± 0.01 μM for EGFR and BRAF^V600E^, respectively.

### 1.1. Rational design

Lapatinib V (Fig. 2) is a highly effective inhibitor that targets both EGFR and HER2. It was authorized by the FDA in 2007 for use in combination with vinorelbine to treat metastatic breast cancer that is HER2-positive.^41^ Reports indicate that lapatinib requires dissolution as a tosylate salt due to its low water solubility. Therefore, clinical applications use lapatinib ditosylate. Meanwhile, the treatment of breast cancer has revealed the adverse effects of lapatinib ditosylate, including gastrointestinal reactions and arrhythmia. Because of these problems, researchers developed new EGFR/HER-2 dual inhibitors that fight tumors, have fewer side effects, and dissolve better in water. Researchers have explored a variety of quinoline compounds with EGFR inhibitory activity.

**Fig. 4:**
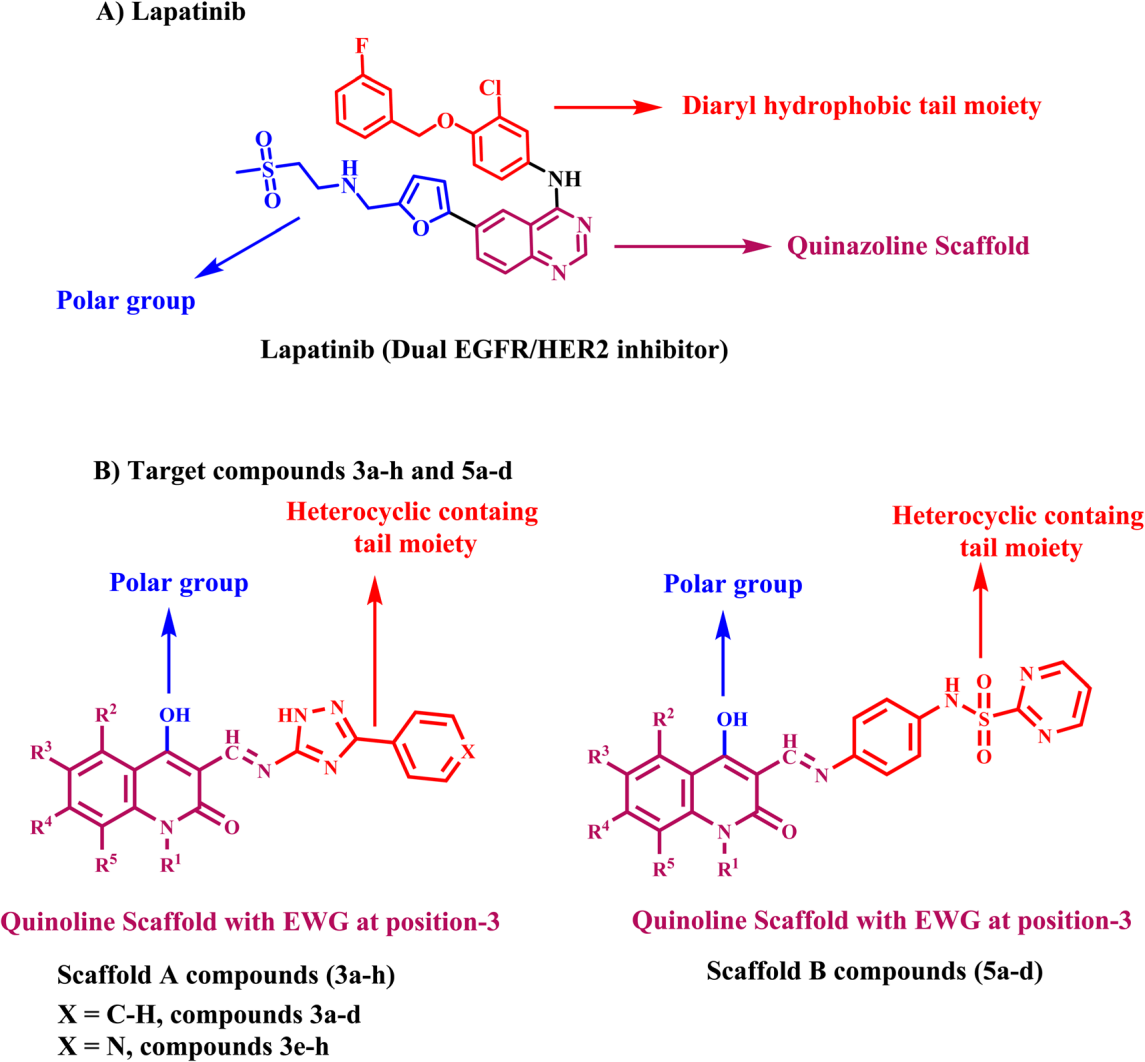
Rational design of (A) lapatinib; (B) target compounds 3a–h and 5a–d.

On the other hand, Weissner *et al.* demonstrated that the N-3 position of the quinazoline ring could be replaced with a C–X, where X represents an electron-withdrawing group.^42^ This study presents the development, synthesis, and biological investigation of new dual inhibitors targeting EGFR and HER-2. We selected lapatinib as the lead compound for these inhibitors. The plan includes assembling a quinoline core scaffold with an Azomethine (Schiff base) group at position 3 and a hydrophobic tail with a heterocyclic structure. The hydrophobic tail can be either a 1,2,4-triazole moiety (Compounds 3a–h, Scaffold A) or a phenyl-pyrimidine-2-sulphonamide moiety (Compounds 5a–e, Scaffold B), Fig. 4.

The newly synthesized compounds 3a–h and 5a–d will be evaluated for their antiproliferative activity against a panel of four cancer cell lines. The most promising compounds will be further investigated as dual EGFR/HER2 inhibitors. Moreover, the apoptotic potential activity of the most potent compounds will be investigated. Finally, we will perform molecular docking analysis to determine these drugs' potential binding mechanisms and interactions with receptor sites.

## 2. Experimental

### 2.1. Chemistry

General Information: see Appendix A (ESI file†).

Starting materials: all 4-hydroxy-2-oxo-1,2-dihydroquinoline-3-carbaldehydes 1a–d were synthesized according to the literature.^43^ Also, 3-aryl-1*H*-1,2,4-triazole-5-amines 2a and 2b,^44^ and *N*-(4-aminophenyl)pyrimidine-2-sulfonamide (4)^45^ were prepared according to reported literature.

#### 2.1.1. General procedure for the formation of compounds 3a–h and 5a–d

In a 250 ml round-bottom flask, 1 mmol of 4-hydroxy-2-oxo-1,2-dihydroquinoline-3-carbaldehydes 1a–d and 160 mg (1 mmol) of 3-phenyl-1*H*-1,2,4-triazol-5-amines (2a), 161 mg (1 mmol) of 3-(pyridin-4-yl)-1*H*-1,2,4-triazol-5-amine (2b) or 250 mg (1 mmol) of *N*-(4-aminophenyl)pyrimidine-2-sulfonamide (4) were dissolved in 30 ml absolute ethanol and refluxed for 5 h with stirring. After the reaction completion, the precipitate was filtered off and washed with hot ethanol three times to afford the corresponding Schiff products 3a–h and 5a–e in excellent yields.

##### 2.1.1.1. (*E*) 4-hydroxy-3-(((3-phenyl-1*H*-1,2,4-triazol-5-yl)imino)methyl)quinolin-2(1*H*)-one (3a)

This compound was found as yellow crystals (EtOH), M.p. 310–12 °C; ^1^H NMR (DMSO-*d*_6_): *δ*_H_ = 13.63 ppm (s, 1H, OH), 12.86 (s, 1H, quinoline-NH), 11.10 (s, 1H, triazole-NH), 9.08 (s, 1H; CH=N), 8.05–7.11 (m, 9H, Ph-H and quinoline-H); ^13^C NMR (DMSO-*d*_6_): *δ*_C_ = 163.12 (C-2), 161.74 (C-4), 157.11 (C-3′), 152.54 (C-5′), 151.74 (CH=N), 141.53 (C-8a), 140.87 (Ar-C), 134.54 (C-7), 134.18 (C-6), 130.86 (C-7), 129.19, 126.16, 125.81 (Ar-CH), 120.20 (C-5), 118.44 (C-8), 91.20 ppm (C-3). *m*/*z* = 331; *Anal. Calcd. For* C_18_H_13_N_5_O_2_: C, 65.25; H, 3.95; N, 21.14. Found: C, 65.35; H, 4.08; N, 20.99.

##### 2.1.1.2. (*E*) 4-hydroxy-6-methyl-3-(((3-phenyl-1*H*-1,2,4-triazol-5-yl)imino)methyl)quinolin-2(1*H*)-one (3b)

This compound was found as yellow crystals (EtOH), M.p. 325–27 °C; ^1^H NMR (DMSO-*d*_6_): *δ*_H_ = 13.64 ppm (s, 1H, OH), 12.86 (s, 1H, quinoline-NH), 11.03 (s, 1H, triazole-NH), 9.07 (s, 1H; CH=N), 8.04(d, *J* = 5.1 Hz, 2H, H-7,8), 7.76 (s, 1H; H-5), 7.56–7.08 (m, 5H, Ph-H), 2.33 ppm (s, 3H; CH_3_); ^13^C NMR (DMSO-*d*_6_): *δ*_C_ = 163.48 (C-2), 162.20 (C-4), 156.84 (C-3′), 152.68 (C-5′), 151.20 (CH=N), 139.46 (C-6), 137.22 (Ar-C), 135.41 (C-7), 132.17 (C-5), 131.75 (C-8a), 128.75, 127.34, 125.64 (Ar-CH), 122.39 (C-4a), 118.16 (C-8), 95.20 (C-3), 21.12 ppm (CH_3_). *m*/*z* = 345; *Anal. Calcd. For* C_19_H_15_N_5_O_2_: C, 66.08; H, 4.38; N, 20.28. Found: C, 65.91; H, 4.17; N, 20.11.

##### 2.1.1.3. (*E*)-4-hydroxy-6-methoxy-3-(((3-phenyl-1*H*-1,2,4-triazol-5-yl)imino)methyl) quinolin-2(1*H*)-one (3c)

This compound was found as yellow crystals (EtOH), M.p. 330–32 °C; ^1^H NMR (DMSO-*d*_6_): *δ*_H H_ = 13.57 ppm (s, 1H, OH), 12.97 (s, 1H, quinoline-NH), 11.00 (s, 1H, triazole-NH), 9.08 (s, 1H; CH=N), 8.04 (t, *J* = 2.4, 5.7 Hz, 2H; Ph-H-o), 7.57–7.12 (m, 6H, Ph-H and quinoline-H), 3.75 ppm (s, 3H; OCH_3_). *m*/*z* = 361; *Anal. Calcd. For* C_19_H_15_N_5_O_3_: C, 63.15; H, 4.18; N, 19.38. Found: C, 63.22; H, 4.09; N, 19.47.

##### 2.1.1.4. (*E*)-4-hydroxy-1-methyl-3-(((3-phenyl-1*H*-1,2,4-triazol-5-yl)imino)methyl)quinolin-2(1*H*)-one (3d)

This compound was found as yellow crystals (EtOH), M.p. 295–297 °C; ^1^H NMR (DMSO-*d*_6_): *δ*_H H_ = 13.70 ppm (s, 1H, OH), 11.00 (s, 1H, triazole-NH), 9.03 (s, 1H; CH=N), 8.01 (d, *J* = 10.5 Hz, 2H, Ph-H-o), 7.62–7.17 (m, 7H, Ph-H, quinoline-H), 3.45 ppm (s, 3H; N–CH_3_); ^13^C NMR (DMSO-*d*_6_): *δ*_C_ = 162.24 (C-2), 158.42 (C-4), 156.79 (C-3′), 153.58 (C-5′), 152.24 (CH=N), 148.58 (C-8a), 135.39 (Ar-C), 134.30 (C-7), 130.16 (C-5), 129.32 (C-6), 129.32, 128.67, 126.24 (Ar-CH), 122.41 (C-4a), 118.82 (C-8), 90.53 (C-3), 20.87 (CH_3_). *m*/*z* = 345; *Anal. Calcd.* For C_19_H_15_N_5_O_2_: C, 66.08; H, 4.38; N, 20.28. Found: C, 66.17; H, 4.22; N, 20.35.

##### 2.1.1.5. (*E*)-4-hydroxy-3-(((3-(pyridin-4-yl)-1*H*-1,2,4-triazol-5-yl)imino)methyl)quinolin-2(1*H*)-one (3e)

This compound was found as yellow crystals (EtOH), M.p. 327–29 °C; ^1^H NMR (DMSO-*d*_6_): *δ*_H_ = 13.56 ppm (s, 1H, OH), 12.86 (s, 1H, quinoline-NH), 11.06 (s, 1H, triazole-NH), 8.98 (s, 1H; CH=N), 8.74 (d, *J* = 5.1 Hz, 2H; pyridine-H-3′′), 7.91 (d, *J* = 4.8 Hz, 2H; pyridine-H-2′′), 7.53 (m, 2H; quinoline-H), 7.12 ppm (m, 2H; quinoline-H). *m*/*z* = 332; *Anal. Calcd.* For C_17_H_12_N_6_O_2_: C, 61.44; H, 3.64; N, 25.29. Found: C, 61.35; H, 3.77; N, 25.41.

##### 2.1.1.6. (*E*)-4-hydroxy-6-methyl-3-(((3-(pyridin-4-yl)-1*H*-1,2,4-triazol-5-yl)imino)methyl) quinolin-2(1*H*)-one (3f)

This compound was found as yellow crystals (EtOH), M.p. 336–38 °C; ^1^H NMR (DMSO-*d*_6_): *δ*_H_ = 13.61 ppm (s, 1H, OH), 12.92 (s, 1H, quinoline-NH), 11.01 (s, 1H, triazole-NH), 9.01 (s, 1H; CH=N), 8.76 (d, *J* = 4.8 Hz, 2H; pyridine-H-3′′), 7.93 (d, *J* = 6.3 Hz, 2H; pyridine-H-2′′), 7.73 (s, 1H; quinoline-H-5), 7.39 (d, *J* = 7.8 Hz, 1H; quinoline-H-7), 7.09 (d, *J* = 7.8 Hz, 1H; quinoline-H-8), 2.31 ppm (s, 3H, CH_3_). *m*/*z* = 346; *Anal. Calcd.* For C_18_H_14_N_6_O_2_: C, 62.42; H, 4.07; N, 24.27. Found: C, 62.38; H, 3.99; N, 24.33.

##### 2.1.1.7. (*E*)-4-hydroxy-6-methoxy-3-(((3-(pyridin-4-yl)-1*H*-1,2,4-triazol-5-yl)imino)methyl) quinol-in-2(1*H*)-one (3g)

This compound was found as yellow crystals (EtOH), M.p. 348–50 °C; ^1^H NMR (DMSO-*d*_6_): *δ*_H_ = 13.57 ppm (s, 1H, OH), 12.99 (s, 1H, quinoline-NH), 10.95 (s, 1H, triazole-NH), 8.99 (s, 1H; CH=N), 8.75 (d, *J* = 8.1 Hz, 2H; pyridine-H-3′′), 7.91 (s, 1H; quinoline-H-5), 7.36 (d, *J* = 6.3 Hz, 2H; pyridine-H-2′′), 7.14 (d, *J* = 9 Hz, 2H; quinoline-H-7,8), 3.77 ppm (s, 3H, OCH_3_). *m*/*z* = 362; *Anal. Calcd.* For C_18_H_14_N_6_O_3_: C, 59.67; H, 3.89; N, 23.19. Found: C, 59.82; H, 3.71; N, 23.33.

##### 2.1.1.8. (*E*)-4-hydroxy-1-methyl-3-(((3-(pyridin-4-yl)-1*H*-1,2,4-triazol-5-yl)imino)methyl) quinolin-2(1*H*)-one (3h)

This compound was found as yellow crystals (EtOH), M.p. 310–12 °C; ^1^H NMR (DMSO-*d*_6_): *δ*_H_ = 12.74 ppm (s, 1H, OH), 10.05 (s, 1H, triazole-NH), 9.05 (s, 1H; CH=N), 8.75 (d, *J* = 4.8 Hz, 2H; pyridine-H-3′′), 7.91 (d, *J* = 4.5 Hz, 2H; pyridine-H-2′′), 7.76–7.21 (m, 4H; quinoline-H), 3.48 ppm (s, 3H, N–CH_3_). *m*/*z* = 346; *Anal. Calcd.* For C_18_H_14_N_6_O_2_: C, 62.42; H, 4.07; N, 24.27. Found: C, 62.56; H, 4.11; N, 24.13.

##### 2.1.1.9. *N*-(4-(((4-hydroxy-2-oxo-1,2-dihydroquinolin-3-yl)methylene)amino)phenyl) pyrimidine-2-sulfonamide (5a)

This compound was found as yellow crystals (EtOH), M.p. 317–19 °C; ^1^H NMR (DMSO-*d*_6_): *δ*_H_ = 13.23 ppm (s, 1H, OH), 11.62 (quinoline-NH), 8.91 (SO_2_NH), 8.62 (s, 1H; CH=N), 8.51–7.05 ppm (m, 11H, quinoline-H, Ph-H and pyrimidine-H). *m*/*z* = 421; *Anal. Calcd. For* C_20_H_15_N_5_O_4_S: C, 57.00; H, 3.59; N, 16.62. Found: C, 56.93; H, 3.66; N, 16.75.

##### 2.1.1.10. *N*-(4-(((4-hydroxy-6-methyl-2-oxo-1,2-dihydroquinolin-3-yl)methylene)amino) phenyl)pyrimidine-2-sulfonamide (5b)

This compound was found as yellow crystals (EtOH), M.p. 331–33 °C; ^1^H NMR (DMSO-*d*_6_): *δ*_H_ = 13.62 ppm (s, 1H, OH), 11.85 (quinoline-NH), 8.91(SO_2_NH), 8.62 (s, 1H; CH=N), 8.52 (d, *J* = 5.1 Hz, 2H; pyrimidine-H-4), 8.04–8.01 (dd, *J* = 3, 2.7 Hz, 2H, quinoline-H-7,8), 7.77–7.70 (q, 1H; pyrimidine-H-5), 7.39 (d, *J* = 8.4 Hz, 2H, H-o), 7.01–7.04 (m, 3H; H-*m*, quinoline-H-5), 2.31 ppm (s, 3H, CH_3_). *m*/*z* = 435; *Anal. Calcd.* For C_21_H_17_N_5_O_4_S: C, 57.92; H, 3.93; N, 16.08. Found: C, 58.01; H, 3.88; N, 15.95.

##### 2.1.1.11. *N*-(4-(((4-hydroxy-6-methoxy-2-oxo-1,2-dihydroquinolin-3-yl)methylene)amino) phenyl)pyrimidine-2-sulfonamide (5c)

This compound was found as yellow crystals (EtOH), M.p. 35 052 °C; ^1^H NMR (DMSO-*d*_6_): *δ*_H_ = 13.52 ppm (s, 1H, OH), 11.82 (quinoline-NH), 9.80 (SO_2_NH), 9.77 (s, 1H; CH=N), 8.93 (d, *J* = 12.6 Hz, 2H; pyrimidine-H-4), 8.52–7.00 (m, 8H, Ph-o,m, pyrimidine-H-5, quinoline-CH), 3.59 ppm (s, 3H, OMe). *m*/*z* = 451; *Anal. Calcd.* For C_21_H_17_N_5_O_5_S: C, 55.87; H, 3.80; N, 15.51. Found: C, 55.77; H, 3.98; N, 15.66.

##### 2.1.1.12. N-(4-(((4-hydroxy-1-methyl-2-oxo-1,2-dihydroquinolin-3-yl)methylene)amino) phenyl)pyrimidine-2-sulfonamide (5d)

This compound was found as yellow crystals (EtOH), M.p. 318–20 °C; ^1^H NMR (DMSO-*d*_6_): *δ*_H_ = 13.72 ppm (s, 1H, OH), 9.63 (SO_2_NH), 9.06 (s, 1H; CH=N), 8.52–7.00 (m, 11H, Ph-o, m, pyrimidine-H-5, quinoline-CH), 3.48 ppm (s, 3H, CH_3_). *m*/*z* = 435; *Anal. Calcd.* For C_21_H_17_N_5_O_4_S: C, 57.92; H, 3.93; N, 16.08. Found: C, 57.79; H, 3.87; N, 15.98.

### 2.2. Biology

#### 2.2.1. Cell viability assay

The impact of compounds 3a–h and 5a–d on cell viability was evaluated using the human mammary (MCF-10A) gland epithelium normal cell line. The MTT assay was used to evaluate the cell viability of 3a–h and 5a–d after a four-day incubation period on MCF-10A cells.^46^ Refer to Appendix A for more details.

#### 2.2.2. Antiproliferative assay

The MTT assay^47,48^ was used to evaluate the antiproliferative impact of 3a–h and 5a–d on four human cancer cell lines: HT-29 for colon cancer, Panc-1 for pancreatic cancer, A-549 for lung cancer, and MCF-7 for breast cancer. Erlotinib was employed as a reference. The IC_50_ values for new compounds were obtained through dose–response experiments. The stated values are based on a minimum of two independent experiments, with three replicates per concentration in each experiment. The experimental details can be found in Appendix A (ESI File†).

#### 2.2.3. EGFR inhibitory assay

Compounds 3e, 5a, 5b and 5d were tested for their potential to inhibit EGFR using the EGFR-TK assay^49^ with erlotinib as the reference compound. See Appendix A for more details.

#### 2.2.4. HER2 inhibitory assay

The compounds 3e, 5a, 5b, and 5d were tested to determine their ability to inhibit HER2 using the kinase assay.^50^ Lapatinib was used as the reference compound. For more details, see Appendix A.

## 3. Results and discussion

### 3.1. Chemistry

**Scheme 1:**
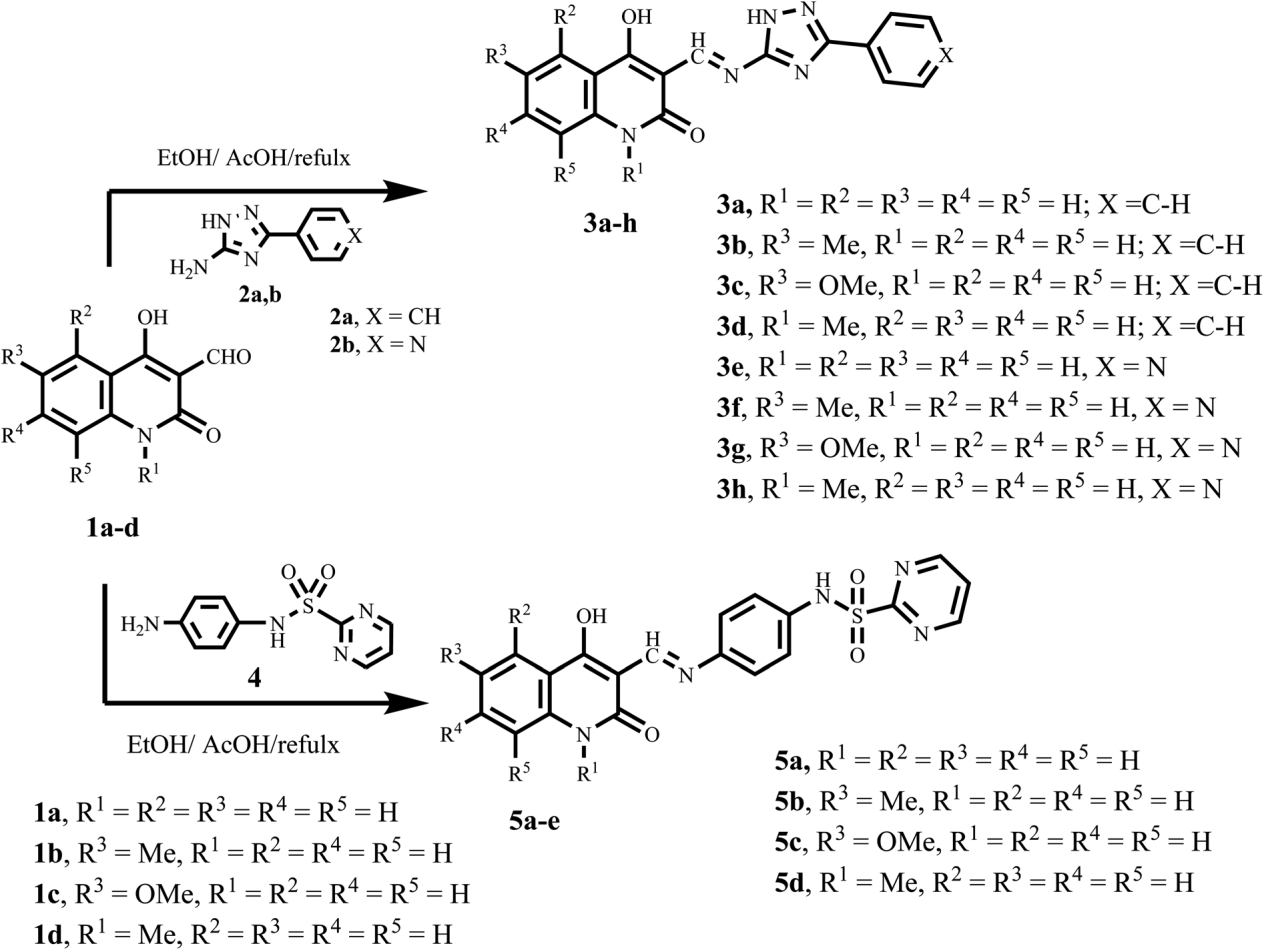
Synthesis of target compounds 3a–h and 5a–d.

We synthesized chalcone compounds by condensing 4-hydroxy-2-oxo-1,2-dihydroquinoline-3-carbaldehydes 1a–f with primary amine derivatives 2a,b, and 4. The condensation of 4-hydroxy-2-oxo-1,2-dihydroquinoline-3-carbaldehyde 1 with 3-phenyl-1*H*-1,2,4-triazol-5-amine (2a) and 3-(pyridin-4-yl)-1*H*-1,2,4-triazol-5-amine (2b) lead to the formation of (*E*) 4-hydroxy-3-(((3-phenyl/or pyridinyl-1*H*-1,2,4-triazol-5-yl)imino)methyl)quinolin-2(1*H*)-ones 3a–h. Moreover, condensation of compound 1 with *N*-(4-aminophenyl)pyrimidine-2-sulfonamide (4) yields *N*-(4-hydroxy-2-oxo-1,2-dihydroquinolin-3-yl)methylene)amino)phenyl)pyrimidine-2-sulfone-amides 5a–e (Scheme 1).

**Fig. 5:**
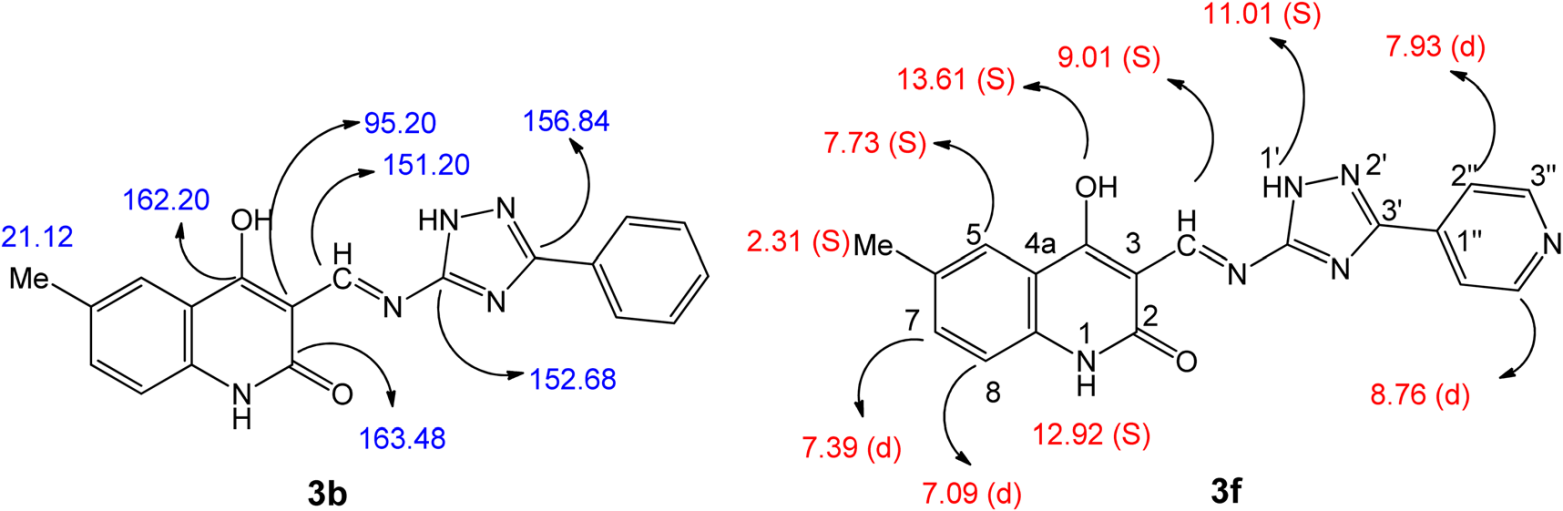
Confirmation structures for compounds 3b and 3f.

All the compounds obtained are the product of a simple and generally recognized condensation process, eliminating the need for additional processes and analyses to confirm their chemical composition. Accordingly, ^1^H NMR, ^13^C NMR spectra, and elemental analysis were used. Compound 3f, namely (*E*)-4-hydroxy-6-methyl-3-(((3-(pyridin-4-yl)-1*H*-1,2,4-triazol-5-yl)imino)methyl)quinolin-2(1*H*)-one (Fig. 5), was chosen for further studies. The ^1^H NMR spectra of 3f has four shielding singlet lines at *δ*_H_ = 13.61, 12.92, 11.01, and 9.01 ppm. These signals are identified as OH, quinoline-NH, triazole-NH, and CH=N groups.

The quinoline-H-5, H-7, and H-8 chemical shifts were seen to be consistent with the reported values ^51–53^ at *δ*_H_ = 7.73 (singlet, 1H), 7.39 (doublet, *J* = 7.8 Hz, 2H), and 7.09 ppm (doublet, *J* = 7.8 Hz, 2H), respectively. Additionally, the pyridinyl group is identified by its 1,4-disubstituted benzene ring structure, which is evident in the ^1^H NMR spectrum as a doublet–doublet pattern at *δ*_H_ of 8.76 ppm (d, *J* = 4.8 Hz, 2H; pyridine-H-3′′) and 7.93 ppm (d, *J* = 6.3 Hz, 2H; pyridine-H-2′′). The ^13^C NMR spectrum of 3b (Fig. 5), (*E*) 4-hydroxy-6-methyl-3-(((3-phenyl-1*H*-1,2,4-triazol-5-yl)imino)methyl)-quinolin-2(1*H*)-one, displays veiled common signals at specified chemical shifts. The signals at *δ*_C_ = 163.48, 162.20, 156.84, 152.68, and 151.20 ppm correspond to C-2, C-4, C-3′, C-5′, and CH=N. There is also a signal at 21.12 ppm, which corresponds to a methyl group.

### 3.2. Biology

#### 3.2.1. Cell viability assay

The effect of new compounds 3a–h and 5a–d on cell viability was evaluated using the MCF-10A (human mammary gland epithelium) normal cell line. The MTT assay was used to evaluate the cell viability of compounds 3a–h and 5a–d after a four-day incubation period on MCF-10A cells.^46^Table 1 demonstrates that none of the compounds examined shown any cytotoxic effects on normal cell, since all compounds exhibited over 85% cell viability at a dose of 50 μM.

**Table 1:** IC_50_ values of compounds 3a–h, 5a–d, and erlotinib against four cancer cell lines

Comp.	Cell viability%	Antiproliferative activity IC_50_ ± SEM (nM)				
		A-549	MCF-7	Panc-1	HT-29	Average (GI_50_)
3a	89	72 ± 7	69 ± 6	74 ± 7	74 ± 7	72
3b	90	70 ± 6	67 ± 6	70 ± 6	71 ± 6	70
3c	87	50 ± 5	46 ± 4	54 ± 5	52 ± 4	51
3d	89	76 ± 7	74 ± 7	76 ± 7	77 ± 7	76
3e	86	32 ± 3	31 ± 3	34 ± 3	34 ± 3	33
3f	90	62 ± 6	59 ± 5	65 ± 6	64 ± 6	63
3g	89	64 ± 6	60 ± 6	64 ± 6	66 ± 6	64
3h	85	82 ± 8	78 ± 7	86 ± 8	82 ± 8	82
5a	87	25 ± 2	23 ± 2	26 ± 2	26 ± 2	25
5b	90	39 ± 4	35 ± 4	41 ± 4	40 ± 3	39
5c	86	53 ± 5	49 ± 4	54 ± 5	55 ± 5	53
5d	90	46 ± 4	42 ± 4	48 ± 4	48 ± 4	46
Erlotinib	ND	30 ± 3	40 ± 3	30 ± 3	30 ± 3	33

#### 3.2.2. Antiproliferative assay

The MTT assay^47,48^ was used to evaluate the antiproliferative impact of compounds 3a–h and 5a–d on four human cancer cell lines: HT-29 for colon cancer, Panc-1 for pancreatic cancer, A-549 for lung cancer, and MCF-7 for breast cancer. Erlotinib was employed as a reference. Table 1 displays the median inhibitory concentration (IC_50_) and average IC_50_ (GI_50_) values for each substance tested on the four cancer cell lines.

Generally, compounds 3a–h and 5a–d had significant antiproliferative activity, with GI_50_ values ranging from 25 to 82 nM when compared to the reference erlotinib (GI_50_ = 33 nM). Furthermore, all evaluated compounds showed greater affinity to the breast cancer (MCF-7) cell line than to the other cell lines tested. Compounds 3e, 5a, 5b, and 5d had the most antiproliferative activity, with GI_50_ values of 33, 25, 39, and 46 nM, respectively. Derivatives 3e, 5a, and 5b outperformed erlotinib against the breast MCF-7 cancer cell line. Their IC_50_ values were 31, 23, and 35 nM, respectively, whereas erlotinib had an IC_50_ value of 40 nM.

Compound 5a (R^1^ = R^2^ = R^3^ = R^4^ = R^5^ = H, Scaffold B) outperformed all of the other compounds tested. It had a GI_50_ of 25 nM, making it 1.3 times more active than erlotinib (GI_50_ = 33 nM) against the four cancer cell lines studied. Compound 5a demonstrated a significant antiproliferative activity against the breast cancer (MCF-7) cell line with an IC_50_ value of 23 nM, which was two times more potent than erlotinib's IC_50_ value of 40 nM. Additionally, compound 5a exhibits a slightly higher potency than erlotinib against the remaining three cell lines, Table 1.

The antiproliferative activity of compounds 3a–h and 5a–d is significantly affected by the substitution pattern at position one (N1) and position six of the quinoline moiety. For example, compound 5d (R^1^ = Me, R^2^ = R^3^ = R^4^ = R^5^ = H, Scaffold B), a derivative with a methyl group linked to the nitrogen atom (N-methyl derivative), was shown to be less efficient as antiproliferative agent than 5a (R^1^ = R^2^ = R^3^ = R^4^ = R^5^ = H, Scaffold B). Compound 5d had a GI_50_ of 46 nM, two times lower than 5a, demonstrating that the presence of a free nitrogen atom at position 1 (N-1) of the quinoline moiety is more tolerated for antiproliferative activity than the N-methyl group. Another example includes the 6-methyl derivative, compound 5b (R^3^ = Me, R^1^ = R^2^ = R^4^ = R^5^ = H, Scaffold B), and the 6-methoxy derivative, 5c (R^3^ = OMe, R^1^ = R^2^ = R^4^ = R^5^ = H, Scaffold B), both of which were revealed to be less effective than the unsubstituted derivative, 5a (R^1^ = R^2^ = R^3^ = R^4^ = R^5^ = H, Scaffold B). Compounds 5b and 5c exhibit IC_50_ values of 39 and 53 nM, respectively, which are 1.6 and 2.2-folds less potent than 5a (GI_50_ = 25 nM). These findings suggest that derivatives with an unsubstituted quinoline moiety are more efficient than derivatives substituted with electron-donating methyl and methoxy groups. However, in order to achieve an appropriate SAR (structure–activity relationship), derivatives of the quinoline moiety's phenyl ring must be substituted with an electron-drawing group such as a halogen atom or nitro group. This precise modification is now being explored in our lab.

Compound 3e (R^1^ = R^2^ = R^3^ = R^4^ = R^5^ = H, X = N, Scaffold A) demonstrated the second highest activity with a GI_50_ value of 33 nM, which is equivalent to the reference erlotinib (GI_50_ = 33). However, 3e exhibited greater activity than Erlotinib against the breast cancer MCF-7 cell line, as shown in Table 1. Substituting the C_6_–H of the quinoline moiety in compound 3e with C_6_-methyl in compound 3f (R^3^ = Me, R^1^ = R^2^ = R^4^ = R^5^ = H, X = N, Scaffold A) or with a methoxy group in compound 3g (R^3^ = OMe, R^1^ = R^2^ = R^4^ = R^5^ = H, X = N, Scaffold A) resulted in a significant decrease in antiproliferative activity. The GI_50_ values for 3f and 3g were 63 and 82 nM, respectively, which were 1.9- and 2.5-fold less potent than 3e (GI_50_ = 33 nM). This supports the notion that the quinoline moiety's unsubstituted phenyl ring was more tolerated for activity.

Moreover, in 3e's pyridine ring, replacing the nitrogen atom with carbon one (phenyl ring) resulted in a confirmed drop in antiproliferative activity. Compound 3a (R^1^ = R^2^ = R^3^ = R^4^ = R^5^ = H, X = CH, Scaffold A) is the phenyl derivative of compound 3e. Its GI_50_ value is 72 nM, making it two times less potent than compound 3e. This indicates that the antiproliferative activity of the 1,2,4-triazole derivatives favors the pyridine ring over the phenyl one.

Finally, it is worth mentioning that compounds 3e and 5a exhibit the most potent antiproliferative activity against all the examined cell lines, particularly the lung cancer A-549 and breast cancer MCF-7 cell lines. Compound 5a exhibited IC_50_ values of 25 and 23 nM against A-549 and MCF-7 cell lines, respectively, making it more efficient than erlotinib against both cell lines (erlotinib's IC_50_ values were 30 and 40 nM, respectively). Compound 3e, the second most active compound, exhibited IC_50_ values of 32 and 31 nM, indicating more potency than erlotinib against the breast MCF-7 cell line. However, it displayed similar potency to erlotinib against the lung A-549 cell line.

#### 3.2.3. EGFR inhibitory assay

**Fig. 6:**
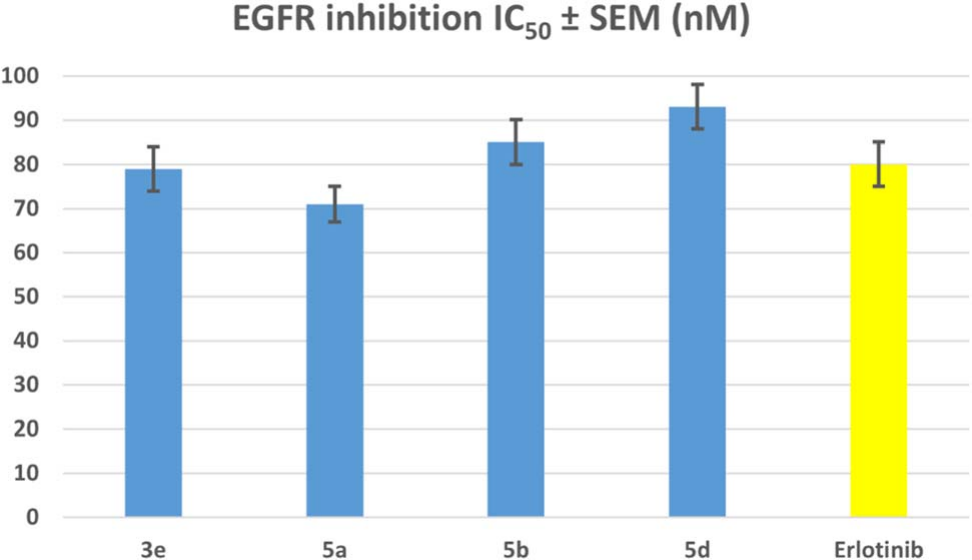
IC_50_ values of compounds 3e, 5a, 5b, 5d, and erlotinib against EGFR.

The most potent antiproliferative derivatives, 3e, 5a, 5b, and 5d, were examined for their potential to impede EGFR through the use of the EGFR-TK assay.^49^ The findings are displayed in Table 2, and Fig. 6. Erlotinib was used as a reference compound.

**Table 2:** IC_50_ values of compounds 3e, 5a, 5b, 5d, erlotinib, and lapatinib against EGFR and HER2

Compound	EGFR inhibition IC_50_ ± SEM (nM)	HER-2 inhibition IC_50_ ± SEM (nM)
3e	79 ± 5	39 ± 2
5a	71 ± 4	31 ± 2
5b	85 ± 5	47 ± 3
5d	93 ± 5	53 ± 3
Erlotinib	80 ± 5	—
Lapatinib	—	26 ± 1

The results of this assay are consistent with the results of the antiproliferative assay, which showed that compounds 5a (R^1^ = R^2^ = R^3^ = R^4^ = R^5^ = H, Scaffold B) and 3e (R^1^ = R^2^ = R^3^ = R^4^ = R^5^ = H, X = N, Scaffold A), the most potent antiproliferative agents, were the most effective derivatives of EGFR inhibitors, with IC_50_ values of 71 ± 4 and 79 ± 5, respectively. Compound 5a exhibited more potency than erlotinib as an EGFR inhibitor, while compound 3e proved comparable efficacy to erlotinib. Compounds 5b and 5d showed significant inhibition of EGFR, with IC_50_ values of 85 and 93 nM, respectively. These compounds had slightly lower potency than erlotinib, Fig. 6. These findings imply that compounds 3e and 5a are highly efficient antiproliferative candidate that may operate as an EGFR inhibitor.

#### 3.2.4. HER2 inhibitory assay

**Fig. 7:**
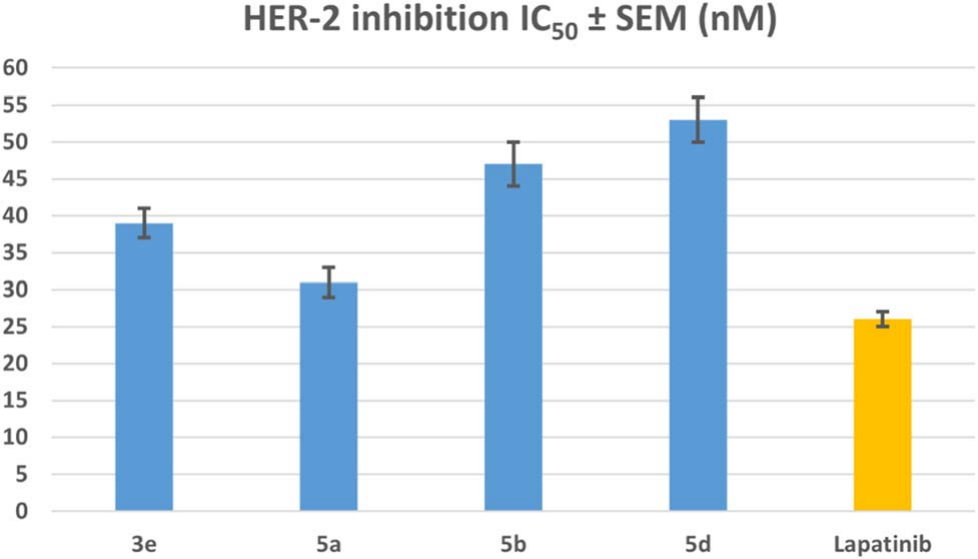
IC_50_ values of compounds 3e, 5a, 5b, 5d, and lapatinib against HER2.

The compounds 3e, 5a, 5b, and 5d were tested to determine their ability to inhibit HER2 using the kinase assay.^50^ The results are presented in Table 2 and Fig. 7. Lapatinib served as the reference compound. The results showed that the compounds tested significantly inhibited HER2, with IC_50_ values ranging from 31 to 53 nM, compared to lapatinib's IC_50_ of 26 nM. In all cases, the tested compounds were less potent than the lapatinib reference drug. Compound 5a was again the most effective HER2 inhibitor, with an IC_50_ value of 31 nM, 1.2 times less potent than lapatinib. These findings revealed that compound 5a is a potential antiproliferative candidate with dual EGFR/HER2 inhibitory activity, prompting structural modification for lead optimization.

#### 3.2.5. Apoptosis assays

Apoptosis is a crucial cell biological process during animal development, tissue maintenance, and immunological responses.^54,55^ However, in normal physiological events in a healthy organism, there is a crucial balance between apoptotic and anti-apoptotic mediators. However, an imbalance may arise in certain situations, which can lead to diseases. Excessive expression or inhibition of apoptotic mediators often causes this imbalance. Pathological conditions such as cancer can revoke this imbalance.^56^

We analyzed compounds 3e and 5a, which showed the highest potency in all laboratory tests, to determine their capacity to trigger the apoptosis cascade and exhibit proapoptotic activity.

##### 3.2.5.1. Caspases-3/8 assays

Cells undergo apoptosis in response to certain signal instructions, leading to major changes. Early in the process, caspases are considered the primary agents of apoptosis and trigger the process. They break down essential cellular elements, such as nuclear proteins like DNA repair enzymes or structural proteins in the cytoskeleton, necessary for efficient cellular function. Caspases can stimulate DNases, enzymes that degrade nuclear DNA.^57,58^ Compounds 3e and 5a were evaluated as caspase-3/8 activators against the MCF-7 breast cancer cell line.^59^ The outcomes of this assay are shown in Table 3.

**Table 3:** Apoptotic potentials of compounds 3e and 5a

Compd no.	Caspase-3		Caspase-8		Bax		Bcl-2	
	Conc. (pg ml^−1^)	Fold change	Conc. (ng ml^−1^)	Fold change	Conc. (pg ml^−1^)	Fold change	Conc. (ng ml^−1^)	Fold reduction
3e	587 ± 5	9	1.65 ± 0.20	19	316 ± 3	35	0.84	6
5a	710 ± 6	11	1.70 ± 0.15	21	350 ± 3	39	0.62	8
Staurosporine	465 ± 4	7	1.60 ± 0.10	18	288 ± 2	32	1.00	5
Control	65	1	0.09	1	9	1	5.00	1

In MCF-7 cells, treatment with compound 5a at its IC_50_ concentration significantly increased the expression levels of active caspases 3 and 8. The expression of active caspase-3 was upregulated 11 times, while active caspase-8 increased by 21 times (Table 3). When cells are treated with Compound 3e, the levels of caspase-3 and caspase-8 go up a lot—by 9 and 19 times more, respectively, than when the cells were not treated. In all cases, compounds 3e and 5a were more effective as caspase-3 and 8 activators than the reference Staurosporine.

##### 3.2.5.2. Proapoptotic BAX and anti-apoptotic Bcl2 assays

The present investigation treated breast (MCF-7) cancer cell lines with compounds 3e and 5a at IC_50_ values. This resulted in a significant upregulation of pro-apoptotic Bax expression levels, with a fold rise of 35 for compound 3e and 39 for compound 5a. Additionally, the treatment led to a notable reduction in anti-apoptotic Bcl-2 expression levels, with a fold decrease of roughly 6 for compound 3e and 8 for compound 5a. These findings are summarized in Table 3. Compounds 3e and 5a markedly elevated the Bax/Bcl-2 ratio compared to the control untreated cells.

### 3.3. Docking study into EGFR

**Fig. 8:**
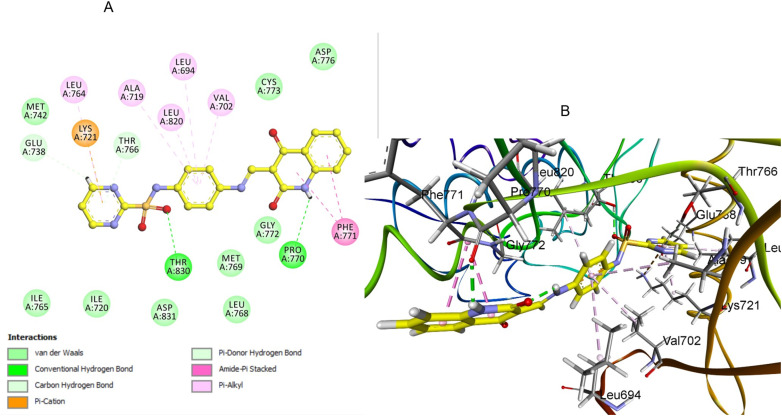
Docking representation models of compound 5a within the binding site of EGFR; (A) 2D-docked model of compound 5a; (B) 3D-docked model of compound 5a.

A comprehensive computational docking analysis was conducted to explore the binding interactions between compounds 3e, 5a, 5b, and 5d with EGFR. The docking study utilized the Discovery Studio software to elucidate the interaction mechanisms of these compounds, ^60^ leveraging the crystallographic structure of the EGFR-erlotinib complex (PDB ID: 1M17) as a structural template. ^61^ The OPLS-AA (Optimized Potentials for Liquid Simulations – All Atom) force field was employed during the energy minimization phase of the molecular systems under investigation. This force field was instrumental in achieving conformational stability of the molecular structures, thereby enhancing the precision and dependability of computational analysis. A comprehensive preparation process was carried out to ensure the accuracy of the protein structure prior to docking. This included careful protein protonation, which further contributed to the reliability of the ensuing docking studies. To validate the docking procedure's effectiveness, the co-crystallized ligand erlotinib was re-docked into the EGFR protein's active site. This re-docking process produced an S score of −7.35 kcal mol^−1^, confirming the accuracy of the docking protocol. The successful outcome was characterized by a key hydrogen bond interaction between the pyrimidine nitrogen of erlotinib and the Met769 residue in the EGFR structure. This interaction is critical for stabilizing the ligand within the active site, underscoring the significance of such molecular interactions in the binding process. Additionally, the analysis of docking scores showed a correlation with *in vitro* EGFR activity levels among the tested hybrid compounds, further validating the docking procedure. Compound 5a exhibited a highly favorable binding pose (−7.53 kcal mol^−1^) within the ATP-binding pocket of EGFR. The quinoline ring forms strong hydrogen bonding with crucial Pro770 residue (Fig. 8). Also, the quinoline ring engaged in strong π–π stacking interactions with aromatic residue Phe771. The pyrimidine sulfonamide moiety participates in Pi-cation interactions with key residue Lys 721 (Fig. 8). The sulfonamide group also engages in hydrogen bonding interactions with Thr830, further stabilizing the binding. The unsubstituted quinoline ring allows for optimal planarity and interaction within the hydrophobic pocket, which is consistent with the SAR finding that unsubstituted derivatives are more active.

**Fig. 9:**
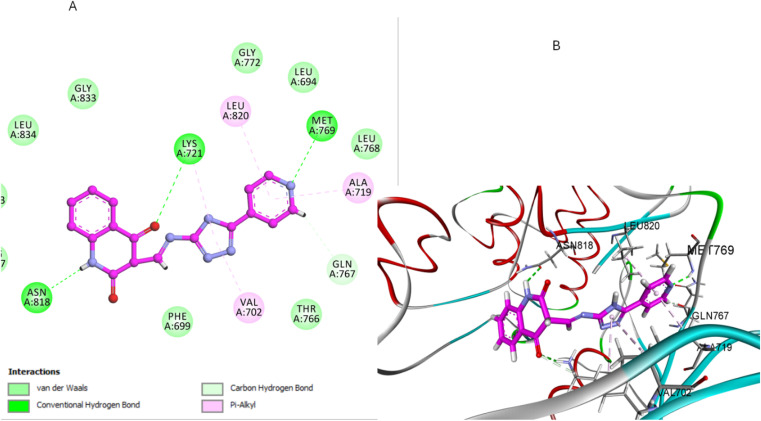
Docking representation models of compound 3e within the binding site of EGFR; (A) 2D-docked model of compound 3e; (B) 3D-docked model of compound 3e.

Similarly, compound 3e, featuring a pyridine-substituted triazole moiety, demonstrated a favorable binding pose (−7.04 kcal mol^−1^) within the EGFR active site. The pyridine ring's nitrogen atom enhances binding by forming a crucial hydrogen bond with a MET769, and the quinoline nitrogen engages in effective H-bonding with ASN818 residue (Fig. 9). As a result, the combination of the pyridine-substituted triazole and the unsubstituted quinoline ring allows 3e to maximize its binding interactions within the pocket, leading to a strong docking score and high binding affinity (Fig. 9).

**Fig. 10:**
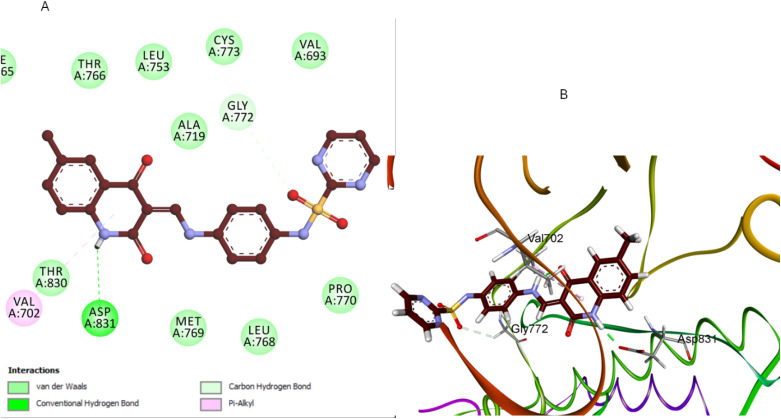
Docking representation models of compound 5b within the binding site of EGFR; (A) 2D-docked model of compound 5b; (B) 3D-docked model of compound 5b.

Compound 5b, which includes a methyl group at position 6 of the quinoline ring, exhibited a moderately favorable binding pose (−6.31 kcal mol^−1^). However, the methyl group introduces steric hindrance that slightly disrupts the optimal interaction between the quinoline ring and the hydrophobic pocket. This alteration in binding dynamics reduces π–π stacking and hydrophobic interactions—the quinoline ring results in one H-bonding interaction with ASP831 and π-alkyl with Val702 (Fig. 10). The methyl group also affects the orientation of the pyrimidine sulfonamide moiety, leading to a less bonding pattern than 5a. The docking results are consistent with the SAR observation that methyl substitution at position 6 of the quinoline ring reduces antiproliferative activity. The steric hindrance introduced by the methyl group in 5b disrupts key interactions within the binding pocket, decreasing binding affinity and biological activity (Fig. 10).

**Fig. 11:**
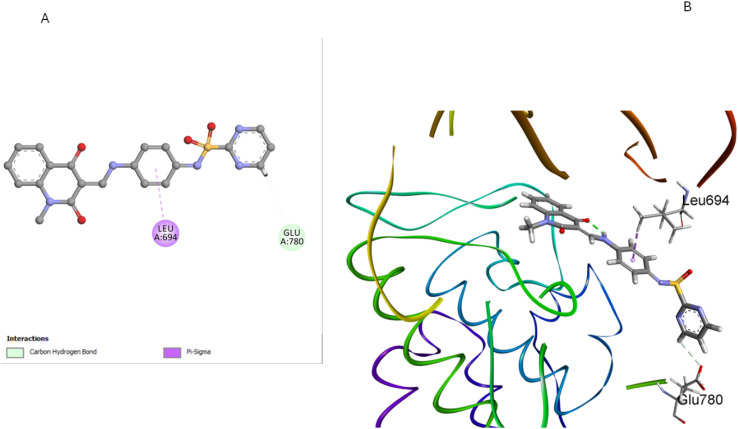
Docking representation models of compound 5d within the binding site of EGFR; (A) 2D-docked model of compound 5d; (B) 3D-docked model of compound 5d.

Compound 5d, which features a methyl group at position 1 of the quinoline ring, showed the least favorable binding pose (−5.72 kcal mol^−1^) among the compounds studied. The methyl group at position 1 disrupts the planarity of the quinoline ring, reducing its ability to participate effectively in π–π stacking interactions. Additionally, this substitution alters the orientation of the entire molecule within the binding site, leading to suboptimal hydrogen bonding with GLU780 and Pi-sigma interactions with LEU694 residues (Fig. 11). The methyl group also causes the sulfonamide moiety to adopt a less favorable conformation, weakening the overall binding affinity. The docking results corroborate the SAR findings that methyl substitution at position 1 of the quinoline ring is detrimental to antiproliferative activity. Due to this substitution, the disruption of key interactions within the EGFR binding site explains the reduced activity 5d compared to the unsubstituted 5a.

The docking studies yielded significant information on the binding interactions of compounds 5a, 3e, 5b, and 5d with EGFR, providing a clear rationale for the observed SAR trends. Compound 5a, featuring an unsubstituted quinoline ring and pyrimidine sulfonamide moiety, exhibited the most favorable interactions and highest binding affinity, aligning with its superior biological activity. Compound 3e, with its pyridine-substituted triazole moiety, showed robust binding interactions, particularly due to the nitrogen atom in the pyridine ring, which enhances its antiproliferative potency. Conversely, electron-donating groups (Me) at positions 1 or 6 of the quinoline ring, as seen in compounds 5b and 5d, led to reduced binding affinity and antiproliferative activity. These findings underscore the importance of maintaining an unsubstituted quinoline ring and carefully selecting substituents on the triazole and pyrimidine moieties to optimize the binding interactions and enhance the antiproliferative activity of these compounds.

### 3.4. ADME studies

Using the SwissADME tool, we comprehensively analyzed the pharmacokinetic profiles of our compounds, 3e and 5a, compared to the FDA-approved reference drug lapatinib. Our analysis revealed compound 3e exhibits a high GI absorption, a notable advantage over 5a and lapatinib, which shows lower absorption levels (Fig. 22, ESI file†). This finding suggests that 3e has superior oral bioavailability. Regarding BBB permeability, none of the compounds, including lapatinib, were predicted to cross the blood–brain barrier. Furthermore, the analysis confirmed that neither 3e nor 5a is a substrate for P-gp, similar to lapatinib. This characteristic is beneficial as it implies that these compounds are less likely to be effluxed out of cells, potentially leading to higher intracellular concentrations and better therapeutic efficacy. A significant advantage of our compounds over lapatinib lies in their interaction with CYP450 enzymes. Both 3e and 5a do not inhibit major CYP450 enzymes (CYP1A2, CYP2C19, CYP2C9, CYP2D6, CYP3A4), unlike Lapatinib, which inhibits several CYP450 isoforms. The lack of CYP450 inhibition in our compounds reduces the potential for drug–drug interactions, a critical consideration in clinical settings where patients are often on multiple medications. While compounds 3e and 5a show significant promise, continued research and development are essential to fully realizing their potential as therapeutic agents.

### 3.5. SAR analysis


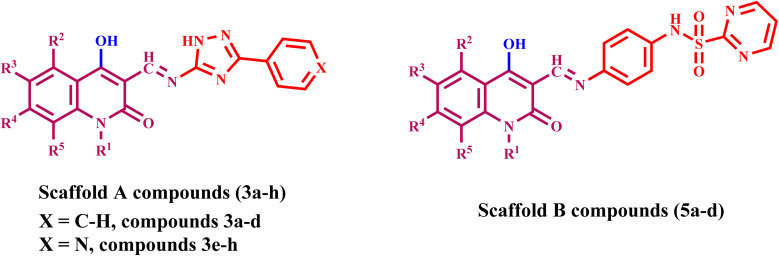
(1) The quinoline moiety in compounds 3a–h and 5a–d is essential for activity. The quinoline ring forms a strong hydrogen bond with the essential Pro770 residue. Additionally, the quinoline ring formed strongπ–π stacking interactions with aromatic residue Phe771.

(2) Scaffold B compounds (5a–d), which contain pyrimidine-2-sulphonamide moiety, are more active than scaffold A compounds (3a–h). The pyrimidine sulfonamide moiety interacts with Lys 721, a critical residue, *via* pi-cation. Moreover, the sulfonamide group engages in hydrogen bonding interactions with Thr830, which further stabilizes the binding.

(3) Among the scaffold A compounds, 3e–h (X = N) are more reactive than 3a–d (X = C–H). In the pyridine-1,2,4-triazole moiety-based derivatives 3e–h, the pyridine ring's nitrogen atom improves binding to EGFR receptors by establishing a hydrogen bond with the crucial MET769 residue, increasing activity.

(4) For compounds 3a–h and 5a–d, the free nitrogen atom (R^1^ = H) at position 1 of the quinoline moiety is essential for activity. The methyl group at position 1 breaks the quinoline ring's planarity, making it less effective in π–π stacking interactions. Furthermore, this replacement changes the overall orientation of the molecule within the binding site.

(5) Substitution at position 6 of the quinoline moiety decreases activity. The unsubstituted quinoline ring provides excellent planarity and interaction within the hydrophobic pocket.

## 4. Conclusion

This study presents a novel class of quinoline compounds, specifically designed as dual inhibitors of EGFR/HER2, suggesting potential anticancer activity. All target compounds underwent preliminary *in vitro* screening against four cancer cell lines. The enzyme and cellular levels revealed compound 5a as the most potent and selective active molecule. Molecular docking experiments also showed that compound 5a had a stable binding to both EGFR and HER-2, with the compound and the enzymes interacting in several ways. Apoptotic markers assays showed that compound 5a raised the levels of apoptotic markers like caspases 3 and 8 and BAX while lowering the levels of the anti-apoptotic Bcl2. The results of this study identified compound 5a as a promising lead, which will be exposed to additional biological assays against several types of breast and lung cancer cell lines, as well as additional research into the mechanism of action, *in vivo* carcinogenic animal models, and lead optimization.

## Data availability

Samples of compounds 3a–h and 5a–d are available from the authors.

## Author contributions

Bahaa G. M. Youssif, Essmat M. El-Sheref, and Hendawy N. Tawfeek: conceptualization, methodology, writing, editing and revision. Safwat M. Rabea: writing, editing and revision. S. Bräse: writing and editing. Hesham A. Abou-Zied: docking analysis and ADMET studies.

## Conflicts of interest

The authors reported no potential conflicts of interest(s).

## Supplementary Material

RA-014-D4RA06394E-s001
